# Pheochromocytoma Crisis Presenting as Cardiac Arrest and Reversible Cardiomyopathy in a Young Adult: A Case Report

**DOI:** 10.7759/cureus.104024

**Published:** 2026-02-21

**Authors:** Eugeniu Gisca, Rui B Dias, Catia R Santos, Luís Val-Flores, Ana Araújo

**Affiliations:** 1 Critical Care Medicine, Unidade Local de Saúde da Região de Leiria - Hospital de Santo André, Leiria, PRT

**Keywords:** adrenal pheochromocytoma, cardiac arrest, neuroendocrine tumor, pheochromocytoma crisis, reversible cardiomyopathy

## Abstract

Acute circulatory collapse and severe myocardial dysfunction in young adults without prior cardiovascular disease represent a critical diagnostic challenge in emergency and intensive care settings. We report the case of a 32-year-old man who presented to the emergency department with acute respiratory failure, profound metabolic acidosis, and hemodynamic instability, rapidly progressing to cardiac arrest with the return of spontaneous circulation. Initial evaluation revealed severe biventricular dysfunction and markedly elevated cardiac biomarkers. CT identified a large right adrenal mass suggestive of pheochromocytoma. Biochemical testing later confirmed markedly elevated plasma and urinary catecholamines and metanephrines. The patient required invasive mechanical ventilation, vasoactive support, and advanced intensive care management. Progressive recovery of myocardial function was observed following stabilization and targeted medical therapy, supporting the diagnosis of catecholamine-induced cardiomyopathy. He was subsequently referred for endocrine follow-up and definitive surgical management. This case highlights pheochromocytoma crisis as a rare but reversible cause of cardiac arrest and acute cardiomyopathy in young adults. Early recognition and coordinated intensive care and endocrine management are crucial to improve outcomes in this potentially fatal condition.

## Introduction

Pheochromocytoma is a catecholamine-secreting neuroendocrine tumor that, although rare, represents a potentially fatal clinical entity when unrecognized [1]. Beyond the classical presentation of hypertension and episodic symptoms, this tumor may precipitate life-threatening cardiovascular emergencies. Pheochromocytoma crisis represents a severe manifestation characterized by catecholamine-induced hemodynamic instability leading to multiple end-organ damage and dysfunction [2]. Despite its clinical relevance, there are no universally accepted diagnostic criteria for pheochromocytoma crisis; the diagnosis is largely based on severe hemodynamic instability, multiorgan dysfunction, and biochemical evidence of catecholamine excess. The condition occurs in approximately 18% of patients with pheochromocytoma and carries a reported mortality rate of 13.8% to 15%, underscoring the critical importance of rapid recognition and urgent intervention [3].

The pathophysiology of cardiac dysfunction in pheochromocytoma is mediated by exaggerated sympathetic stimulation and toxic circulating levels of catecholamines [1]. Excess catecholamine exposure results in a form of stress cardiomyopathy characterized by acute and typically reversible systolic and diastolic dysfunction, with regional wall motion abnormalities extending beyond the distribution of a single coronary artery [1]. The myocardial toxicity of catecholamines includes intracellular calcium overload, coronary vasoconstriction, oxidative stress through free radical generation, and impaired myocardial energy metabolism [4]. Multiple phenotypes of catecholamine-induced cardiomyopathy have been described, including dilated cardiomyopathy, Takotsubo syndrome, inverted Takotsubo variants, and hypertrophic obstructive cardiomyopathy [5].

A distinctive feature of pheochromocytoma crisis is its potential to present with severe cardiovascular collapse. Approximately 2% of patients with pheochromocytoma initially present with cardiogenic shock [1], manifesting as abrupt hemodynamic instability that may progress to cardiac arrest [2,6]. Blood pressure instability in pheochromocytoma crisis can be particularly challenging, with rapid alternation between hypertensive and hypotensive states occurring over seconds to minutes [2]. These presentations may be complicated by malignant ventricular arrhythmias and may require advanced mechanical circulatory support when conventional medical therapy is insufficient [1,2].

Despite the dramatic acute presentation, catecholamine-induced myocardial dysfunction is characteristically reversible. Studies have consistently demonstrated complete recovery of left ventricular systolic function following appropriate medical stabilization and tumor-directed therapy, with significant improvement often occurring within weeks [5]. Early intensive care management and adequate alpha-adrenergic blockade before surgical resection are key elements in preventing irreversible myocardial injury and adverse cardiac remodeling [6].

Pheochromocytoma crisis may be precipitated by several triggering factors, including severe physiological stress such as infection, trauma, pain, or childbirth, as well as iatrogenic causes such as abrupt withdrawal of alpha-blockers or exposure to contrast media [1]. The absence of uniform diagnostic criteria and the wide spectrum of clinical presentations, ranging from hypertensive emergencies to cardiogenic shock and multiorgan failure, contribute to frequent diagnostic delay [1]. Given its rarity and potential for rapid deterioration, pheochromocytoma should be considered in the differential diagnosis of young patients presenting with unexplained shock, acute cardiac dysfunction, or life-threatening arrhythmias [2,6].

In this context, we present a case of pheochromocytoma crisis in a young adult who developed acute cardiovascular collapse and cardiac arrest in the absence of previously known cardiovascular disease. This case is reported to illustrate an uncommon but clinically relevant presentation of pheochromocytoma, highlighting the diagnostic complexity and the need for a high index of suspicion in young patients presenting with unexplained hemodynamic instability and cardiac arrest in the acute care setting.

## Case presentation

A 32-year-old man with no known past medical history or previously diagnosed cardiovascular disease was brought to the emergency department with acute respiratory distress and rapidly progressive clinical deterioration. On admission, he was critically ill, presenting with marked hemodynamic instability, with labile blood pressure values alternating between hypertensive episodes (systolic blood pressure >200 mmHg) and hypotensive states (systolic blood pressure <90 mmHg), associated with tachycardia (heart rate: 110 beats per minute), tachypnea (respiratory rate: approximately 25 breaths per minute), and severe hypoxemia, with peripheral oxygen saturation of 60% despite high-concentration oxygen therapy via a non-rebreather mask (15 L/minute).

Physical examination revealed profuse diaphoresis, cold extremities, altered mental status (Glasgow Coma Scale score of 13), and severe respiratory distress with signs of respiratory muscle fatigue. Purulent sputum was noted, and lung auscultation revealed diffuse bilateral crackles. No focal neurological deficits or peripheral oedema were observed.

Arterial blood gas analysis at admission demonstrated acidemia with metabolic acidosis (pH: 7.22, pCO₂: 40 mmHg, bicarbonate: 16.4 mEq/L), severe hypoxemia (pO₂: 43 mmHg, oxygen saturation: 66%), and marked hyperlactatemia (lactate: 6.6 mmol/L). Chest radiography showed diffuse bilateral alveolar infiltrates, without evidence of pleural effusion.

Shortly after arrival, the patient suffered a cardiac arrest. The initial documented rhythm was pulseless electrical activity (non-shockable rhythm). Advanced life support was immediately initiated, including chest compressions, administration of 1 mg intravenous epinephrine, and endotracheal intubation. Approximately two minutes later, rhythm analysis revealed ventricular fibrillation, and a single defibrillation shock of 200 J was delivered, with the return of spontaneous circulation and restoration of sinus rhythm at a rate of 120-140 beats per minute.

**Figure 1:**
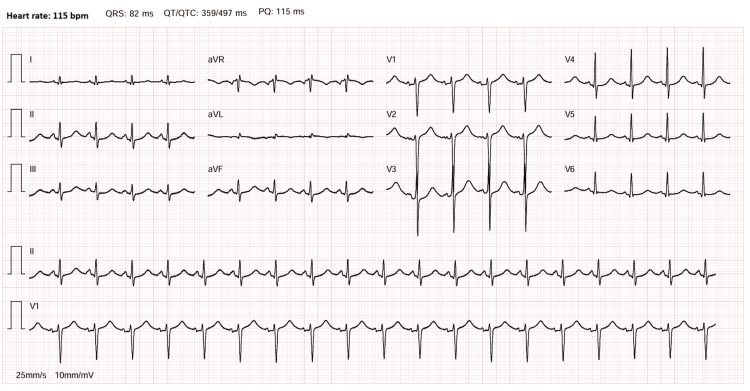
Electrocardiography at admission to the intensive care unit.

Following resuscitation, the patient was admitted to the intensive care unit (ICU) for further stabilization and neuroprotection. Electrocardiography demonstrated sinus rhythm at 115 beats per minute, without evidence of acute ischemic changes or atrioventricular or intraventricular conduction abnormalities (Figure 1).

Initial laboratory evaluation revealed severe myocardial injury, with markedly elevated cardiac biomarkers, including high-sensitivity troponin I (42,388 pg/mL), N-terminal pro-B-type natriuretic peptide (6,230 pg/mL), creatine kinase (1,212 U/L), and creatine kinase-MB (110 U/L). Elevated inflammatory markers were also observed, including C-reactive protein (161 mg/L), procalcitonin (8.5 ng/mL), and leukocytosis (11,400/µL with 85% neutrophils). Initial vital signs and routine laboratory results on admission are summarized in Table 1.

**Table 1:** Vital signs and routine laboratory results on admission at the emergency department. Except for catecholamine metabolites, these were measured after clinical stabilization.

Parameter	Result	Reference range	Units
Vital signs			
Systolic blood pressure	>200 (episodes)/<90 (episodes)	90–120	mmHg
Heart rate	110	60–100	beats/minute
Respiratory rate	~25	12–20	breaths/minute
Peripheral oxygen saturation (SpO2) on a 15 L/minute non-rebreather mask	60	≥94	%
Arterial blood gas			
pH	7.22	7.35–7.45	—
Partial pressure of carbon dioxide (pCO₂)	40	35–45	mmHg
Partial pressure of oxygen (pO₂)	43	80–100	mmHg
Bicarbonate (HCO₃⁻)	16.4	22–26	mEq/L
Oxygen saturation (arterial)	66	95–100	%
Lactate	6.6	0.5–2.0	mmol/L
Routine blood tests			
White blood cell count	11.4	4.0–11.0	×10³/µL
C-reactive protein	161	<5	mg/L
Procalcitonin	8.5	<0.05	ng/mL
Creatine kinase	1,212	30–200	U/L
Creatine kinase-MB	110	<5	U/L
High-sensitivity troponin I	42,388	<34	pg/mL
N-terminal pro–B-type natriuretic peptide	6,230	<125	pg/mL
Catecholamine metabolite			
Plasma metanephrines	3,501	<90	pg/mL
Plasma normetanephrines	4,161	<180	pg/mL
24-hour urinary metanephrines	42,502	<350	µg/24 hours
24-hour urinary normetanephrines	9,373	<600	µg/24 hours

Transthoracic echocardiography revealed severe global biventricular systolic dysfunction with diffuse hypokinesia of both ventricles and markedly reduced cardiac output, in the absence of regional wall motion abnormalities suggestive of acute coronary syndrome, as documented in the formal echocardiographic report.

**Figure 2:**
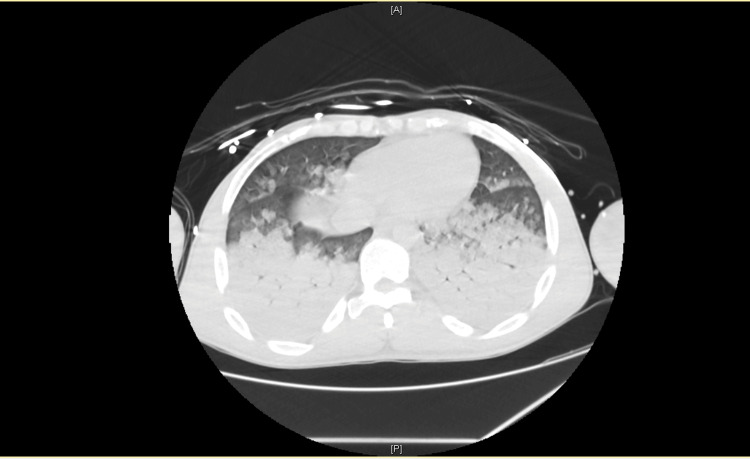
CT revealing large areas of consolidation predominantly affecting the lower lobes.

**Figure 3:**
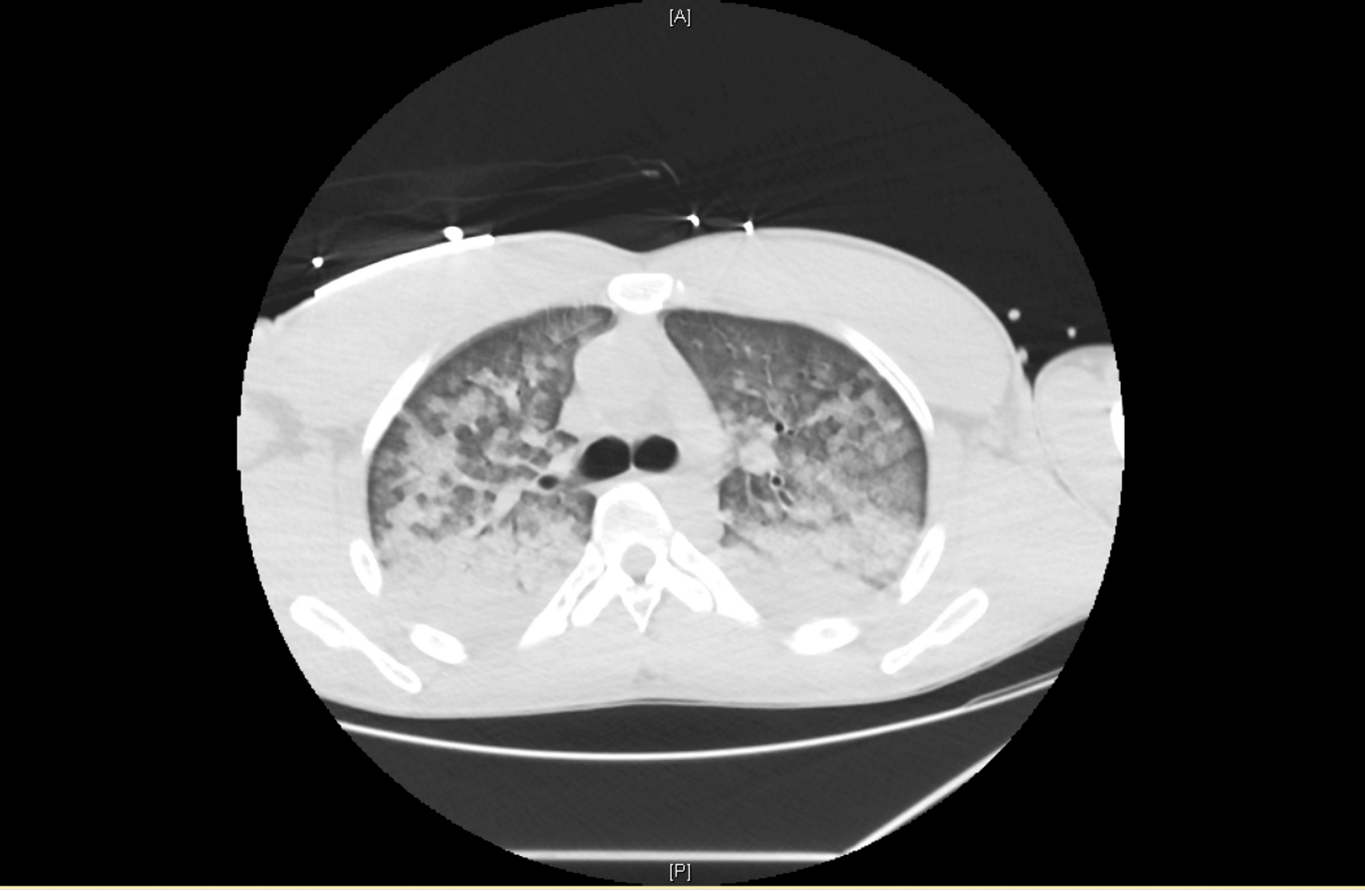
CT showing extensive bilateral pulmonary involvement with widespread ground-glass opacities.

CT performed during the initial diagnostic workup revealed extensive bilateral pulmonary involvement, with large areas of consolidation predominantly affecting the lower lobes (Figure 2), associated with widespread ground-glass opacities involving more than 75% of the remaining lung parenchyma (Figure 3).

**Figure 4:**
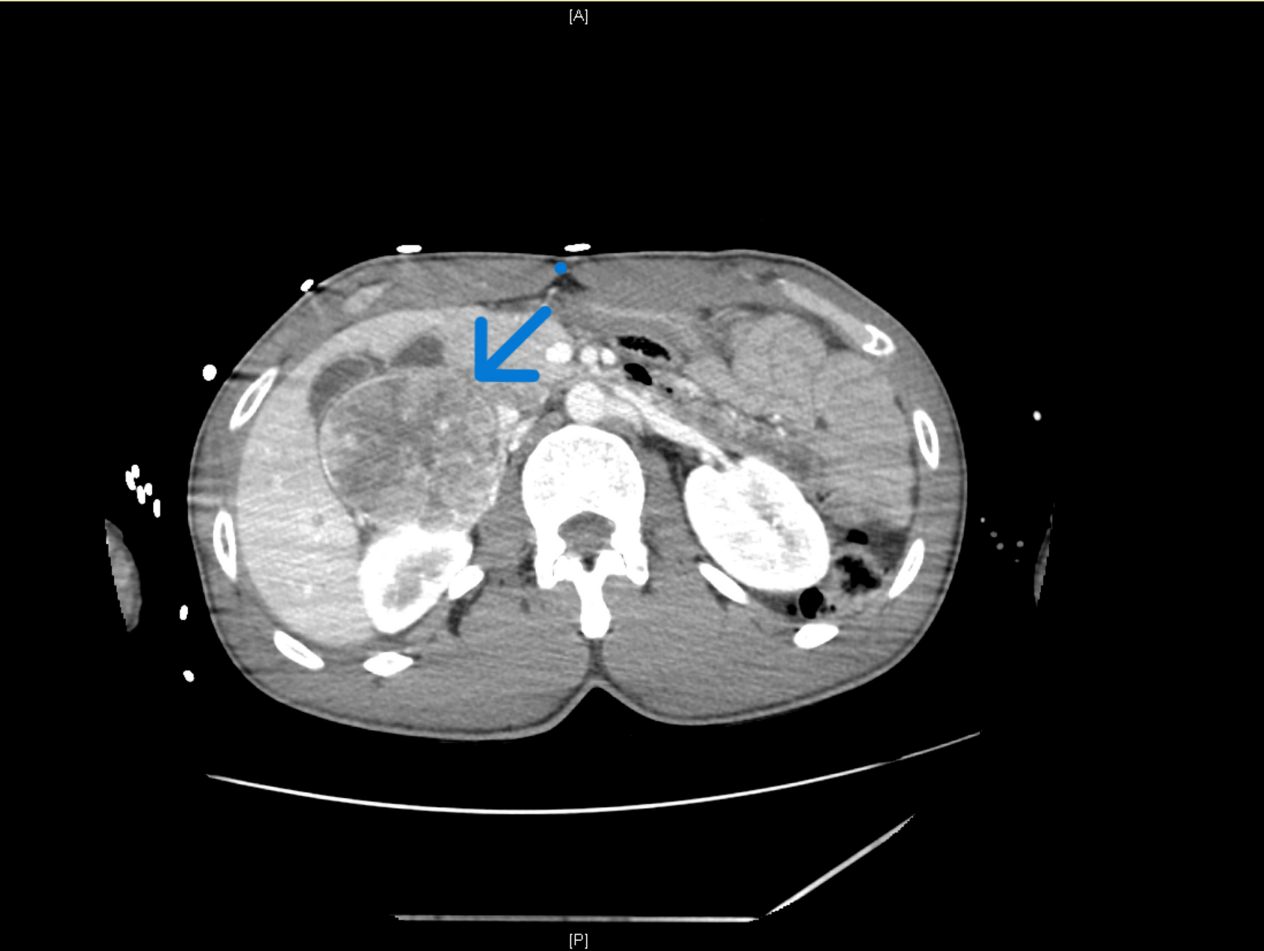
Right adrenal mass. Large right adrenal mass (68 × 68 × 75 mm), markedly hypervascular, without calcifications or evidence of invasion of adjacent structures, findings highly suggestive of pheochromocytoma.

Abdominal imaging identified a large right adrenal mass (Figure 4) measuring approximately 68 × 68 × 75 mm, markedly hypervascular, predominantly at the periphery, without calcifications or evidence of invasion of adjacent structures, findings highly suggestive of pheochromocytoma.

In the context of severe cardiovascular instability and unexplained cardiomyopathy with reduced ejection fraction, pheochromocytoma was strongly suspected. Subsequent biochemical testing confirmed extreme catecholamine excess, with markedly elevated plasma and 24-hour urinary metanephrines (3,501 pg/mL and 42,502 µg/24 hours, respectively) and normetanephrines (4,161 pg/mL and 9,373 µg/24 hours, respectively), far exceeding the upper reference limits. Plasma and urinary catecholamine and metanephrine measurements were obtained after clinical stabilization, in the absence of ongoing vasopressor therapy. These findings supported the diagnosis of a catecholamine-secreting adrenal tumor with an adrenergic biochemical phenotype, complicated by pheochromocytoma crisis and catecholamine-induced cardiomyopathy.

The patient required intensive care support for seven days, including invasive mechanical ventilation, vasopressor and inotropic therapy, and close hemodynamic monitoring. Given the suspicion of concomitant community-acquired pneumonia, empiric antibiotic therapy was initiated with amoxicillin-clavulanate (1.2 g every eight hours for seven days) and azithromycin (500 mg once daily for three days). Over the following days, progressive clinical stabilization was observed, with normalization of acid-base status, resolution of hyperlactatemia, and gradual improvement in myocardial function and oxygen requirements. Repeat echocardiographic assessment demonstrated complete recovery of biventricular systolic function, consistent with reversible catecholamine-induced cardiomyopathy.

After stabilization, the patient was transferred to the medical ward and followed by the Endocrinology and General Surgery teams. Alpha-adrenergic blockade was initiated with doxazosin at a dose of 4 mg once daily and progressively titrated, achieving adequate blood pressure control. After appropriate preoperative preparation, elective surgical resection of the adrenal tumor was performed without perioperative complications. Histopathological examination confirmed the diagnosis of pheochromocytoma.

**Figure 5:**
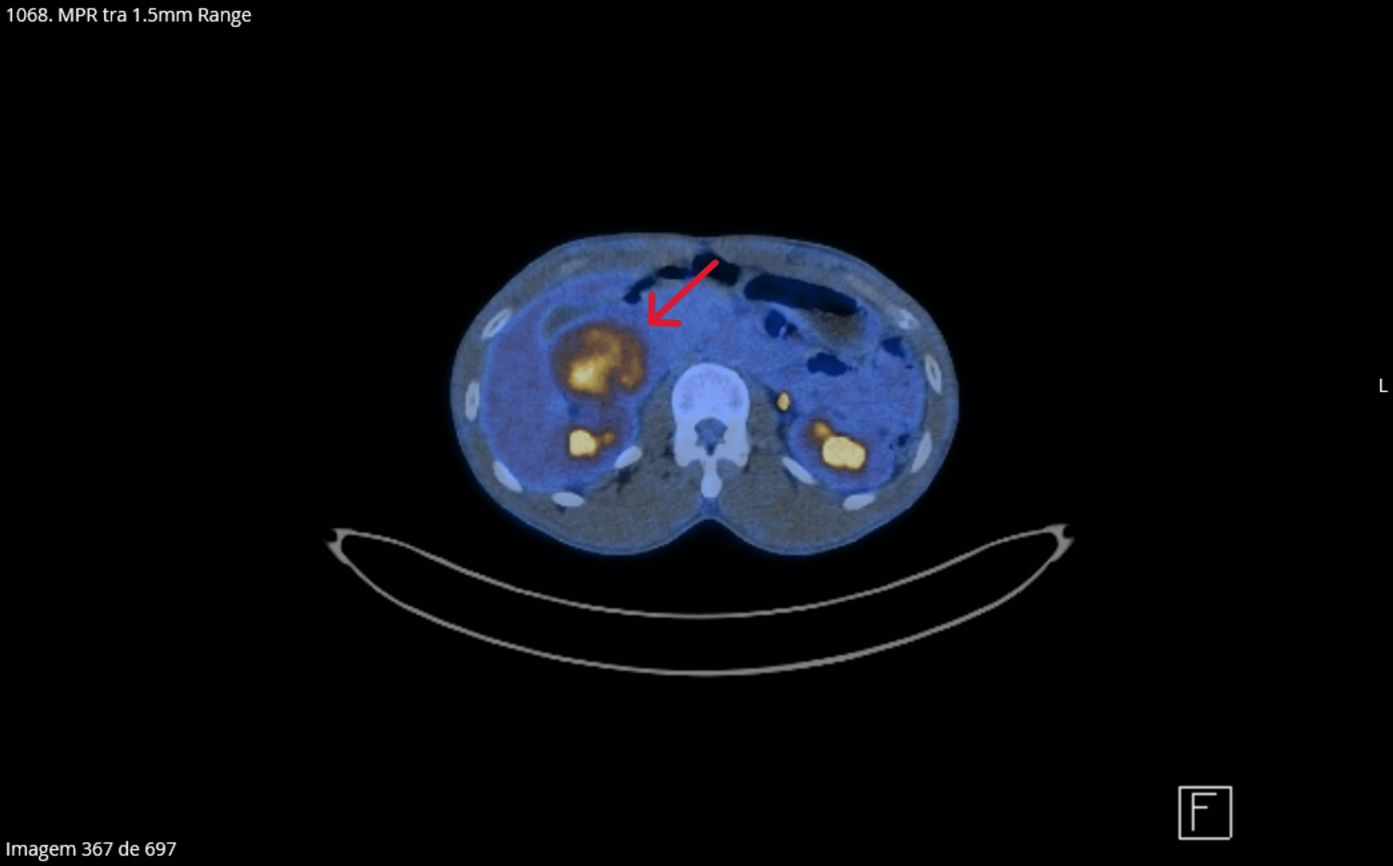
18F-fluorodeoxyglucose positron emission tomography/computed tomography (18F-FDG PET/CT) scan which demonstrates intense heterogeneous FDG uptake of the adrenal mass (SUVmax: 9.2).

Preoperative evaluation included an 18F-fluorodeoxyglucose positron emission tomography/computed tomography (18F-FDG PET/CT) scan, which demonstrated intense heterogeneous FDG uptake of the adrenal mass (SUVmax: 9.2), without evidence of hypermetabolic lymph nodes, pulmonary nodules, skeletal lesions, or distant metastatic disease (Figure 5).

At follow-up, the patient remained clinically well, asymptomatic, and hemodynamically stable, with sustained recovery of cardiac function and no recurrence of cardiovascular symptoms.

## Discussion

This case illustrates a severe presentation of pheochromocytoma crisis, manifesting as cardiac arrest and profound catecholamine-induced cardiomyopathy in a young, previously healthy patient. Pheochromocytoma crisis has been reported in approximately 18% of patients with pheochromocytoma and represents an extreme form of catecholamine excess associated with marked hemodynamic instability and multiorgan dysfunction [1,3]. The abrupt clinical deterioration observed in this patient, culminating in heart failure, malignant arrhythmias, and cardiac arrest, emphasizes the potential for pheochromocytoma to present as a life-threatening cardiovascular emergency, even in the absence of prior clinical history of cardiovascular disease.

The cardiovascular manifestations in this case are consistent with acute catecholamine-induced myocardial toxicity. Excessive catecholamine exposure can result in direct myocardial injury through multiple mechanisms, including intracellular calcium overload, coronary vasoconstriction, oxidative stress, and impaired myocardial energy metabolism [4]. These effects may lead to a spectrum of phenotypes, ranging from Takotsubo-like patterns to dilated cardiomyopathy [5]. In the present case, the combination of markedly elevated cardiac biomarkers, severe global biventricular systolic dysfunction, and the absence of electrocardiographic or echocardiographic features suggestive of acute coronary syndrome supports the diagnosis of catecholamine-induced cardiomyopathy rather than ischemic myocardial injury. Importantly, this form of myocardial dysfunction is typically reversible following adequate medical stabilization and removal of the catecholamine source [5,6].

In addition, the biochemical profile observed in this patient is consistent with an adrenergic biochemical phenotype, characterized by a significant proportion of epinephrine and metanephrine secretion. In contrast to the more common noradrenergic phenotype, which predominantly produces norepinephrine and normetanephrine, adrenergic tumors are frequently associated with kinase signaling-driven pathways and may present with more dramatic cardiovascular instability and perioperative complications. Despite the severe acute presentation, this phenotype has been associated with a lower long-term metastatic risk compared with other molecular subtypes. This biochemical characterization may partly explain the abrupt cardiovascular collapse observed in our patient while also supporting the favorable postoperative course [7].

Diagnosing pheochromocytoma crisis in the acute care setting remains challenging due to its heterogeneous presentation. Severe hemodynamic instability with extreme blood pressure lability, acute cardiac dysfunction, and unexplained cardiogenic shock in young patients should prompt consideration of catecholaminergic disorders [1]. Although biochemical confirmation with plasma or urinary metanephrines is essential for definitive diagnosis, imaging findings such as a hypervascular adrenal mass may provide early diagnostic clues in critically ill patients [3]. In this case, biochemical confirmation was obtained after initial stabilization and in the absence of vasopressor support, strengthening the diagnostic validity of the findings. In the present case, the markedly elevated plasma and urinary metanephrines and normetanephrines were not only diagnostic but also reflective of the magnitude of catecholamine excess driving the acute cardiovascular collapse. The substantial biochemical elevation supported the prompt initiation and careful titration of alpha-adrenergic blockade and reinforced the decision to delay surgical intervention until hemodynamic stabilization was achieved. Thus, quantitative biochemical assessment played a central role in both confirming the diagnosis and guiding therapeutic strategy. Notably, the adrenal mass identified on abdominopelvic CT scan exhibited imaging features highly suggestive of pheochromocytoma, which, when considered in conjunction with the clinical presentation, strongly supported the diagnosis even before biochemical confirmation was available.

The pulmonary manifestations observed in this patient likely reflect the systemic impact of catecholamine excess, characterized by acute pulmonary edema secondary to severe myocardial dysfunction. Multiorgan involvement is a recognized feature of pheochromocytoma crisis and may complicate the initial clinical picture, contributing to diagnostic delay [1,3]. Severe hypoxia may have further amplified catecholamine synthesis and release, potentially exacerbating the hemodynamic instability observed in this patient. Although concomitant pneumonia may have acted as a precipitating stressor for pheochromocytoma crisis, the extent and rapid reversibility of pulmonary involvement suggest that acute cardiogenic pulmonary edema secondary to severe myocardial dysfunction was the predominant mechanism.

Management of pheochromocytoma crisis requires a multidisciplinary approach involving intensive care, endocrinology, and surgery. Aggressive supportive care in the ICU is essential to stabilize end-organ dysfunction. Adequate alpha-adrenergic blockade remains a cornerstone of treatment to control blood pressure and prevent further catecholamine-mediated complications before surgical resection [3,5]. In this case, initiation and titration of alpha-blockade with doxazosin allowed hemodynamic stabilization and safe planning of elective adrenalectomy, resulting in complete clinical and functional recovery.

Recent advances in the molecular understanding of pheochromocytoma and paraganglioma have highlighted the role of hypoxia-inducible signaling pathways in tumor biology. Hypoxia-inducible factor 2α (HIF2α) has been shown to drive tyrosine hydroxylase expression, the rate-limiting enzyme in catecholamine synthesis, thereby contributing to excessive catecholamine production. Emerging targeted therapies, such as the HIF2α inhibitor belzutifan, have demonstrated rapid normalization of catecholamine levels in selected patients, representing a promising therapeutic strategy in advanced or unresectable disease [8]. Although not applicable in the present case, these developments illustrate the evolving landscape of pheochromocytoma and paraganglioma management.

This case adds to the growing body of evidence supporting pheochromocytoma as a potentially reversible cause of acute cardiomyopathy, cardiogenic shock, and cardiac arrest. It highlights the importance of maintaining a high index of suspicion for pheochromocytoma crisis in young patients presenting with unexplained cardiovascular collapse. Early recognition, appropriate supportive care, and timely endocrine and surgical management are critical to achieving favorable outcomes in this rare but potentially fatal condition.

Given the known heritable nature of pheochromocytoma and paraganglioma, genetic evaluation is recommended in all patients regardless of age or family history. The patient has been referred for germline genetic testing in accordance with current clinical practice recommendations.

## Conclusions

Pheochromocytoma crisis is a rare but potentially fatal condition that may present with abrupt cardiovascular collapse, severe myocardial dysfunction, and cardiac arrest, even in young patients without previous cardiovascular disease. This case highlights pheochromocytoma as a reversible cause of acute cardiomyopathy and cardiogenic shock when promptly recognized and appropriately managed. Early clinical suspicion, combined with targeted imaging, biochemical confirmation, and coordinated multidisciplinary care in the intensive care setting, is essential to achieve favorable outcomes in this life-threatening but treatable condition.
